# Wolfram syndrome type 1: a case series

**DOI:** 10.1186/s13023-023-02938-5

**Published:** 2023-11-16

**Authors:** Danyang Du, Aihemaitijiang Tuhuti, Yanrong Ma, Munila Abuduniyimu, Suli Li, Guoying Ma, Jazyra Zynat, Yanying Guo

**Affiliations:** Department of Endocrinology and Metabolism, People’s Hospital of Xinjiang Uygur Autonomous Region, Xinjiang Clinical Research Center for Diabetes, Urumqi, 830000 China

**Keywords:** Wolfram syndrome, *WFS1* gene, Diabetic mellitus, Genetic testing

## Abstract

**Background:**

Wolfram syndrome (WS) is a rare autosomal recessive multisystem neurodegenerative disease characterized by non-autoimmune insulin-dependent diabetes mellitus, optic atrophy, sensorineural deafness, and diabetes as the main features. Owing to clinical phenotypic heterogeneity, the misdiagnosis rate is high. However, early accurate diagnosis and comprehensive management are key to improving quality of life and prolonging life.

**Results:**

Eleven patients from seven WS pedigrees with 10 mutation sites (c.1314_1317delCTTT, c.C529T, c.C529A, c.G2105A, c.C1885T, c.1859_1860del, c.G2020A, c.C529A, c.G2105A, and c.G1393C) in the *WFS1* gene were included. We conducted further expert department analysis to clarify the diagnosis and analyze the correlation between genes and phenotypes.

**Conclusions:**

The genotypes of these patients were closely associated with their phenotypes. The clinical data of the patients were analyzed to provide a basis for the diagnosis and clinical management of the disease.

## Background

Wolfram syndrome (WS) is a rare autosomal recessive disorder caused by mutations in the *WFS1* or *CISD2* genes, with a global prevalence of one in 500,000 [1]. The patients often present with diabetes mellitus, optic atrophy, diabetes insipidus, sensorineural deafness, abnormal urinary tract function, or neuropsychiatric disorders. Diabetes is often the first clinical symptom observed in children and adolescents. Their average life expectancy is approximately 30 years [2]. The clinical presentation of this disease varies widely, and its diagnosis is challenging. WS presents in two phenocopies, Wolfram syndrome type 1 (WS1) and Wolfram syndrome type 2 (WS2), respectively caused by homozygous mutations of the *WFS1* and *CISD2* genes. The WS1 shows a more severe clinical manifestations and poor prognosis [3]. The existence of the Autosomal Dominant Wolfram-like syndrome (OMIM # 614,296) is caused by heterozygous mutations of the *WFS1* gene. The disease involves multiple systemic organ systems, including the eyes, ears, and kidneys, and a large age span of different clinical features appears, therefore, the condition is highly heterogeneous and the diagnosis is challenging.

## Results

### Clinical presentation

**Fig. 1:**
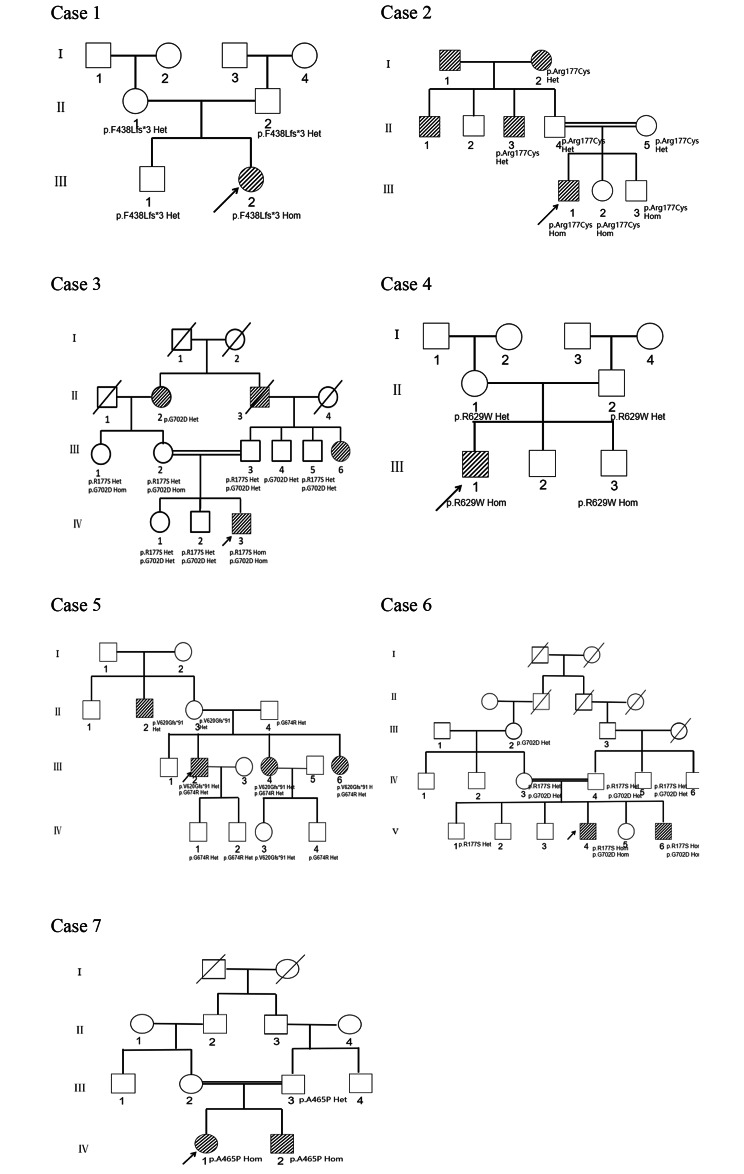
Genealogical trees of seven Wolfram syndrome families. Black-and-white striped circle or square = patients with diabetes; white circle or square = healthy individuals

All patients diagnosed with WS at a tertiary hospital in Xinjiang, China from 2016 to 2022 were included in these case series. Eleven cases were included, and 7 out of the 11 cases were male. The age at WS diagnosis ranged from 8 to 30 years. For a summary of the genealogy, see Fig. 1.

#### Case 1

A 21-year-old female had experienced gradual dry mouth, polyuria, and fatigue over the past 14 years. At the age of 7, because fasting blood glucose was 28.0 mmol/L, the patient was diagnosed with “type 1 diabetes mellitus” and treated with insulin injection. At 19 years of age, the patient developed decreased vision in both eyes. The patient was otherwise healthy and did not take any medications. The patient’s parents denied consanguineous marriage or family history of diabetes. The patient had a BMI of 19.5 kg/m2, an HbA1c rate of 9.8%, double hydronephrosis, bilateral ureteral dilatation, diabetic retinopathy, and optic nerve papillary atrophy in both eyes. Hearing test results were normal. Diagnosis of WS was suspected. The genetic testing identified the c.1314_1317delCTTT,p.F438Lfs * 3 homozygous mutation of the WFS1 gene. Sanger sequencing of family members found both parents and the elder brother being heterozygous carriers of the WFS1 mutation.

#### Case 2

A 14-year-old male noticed dry mouth, polyuria for the past 4 years, and slow growth for the past 3 years. At the age of 10, because fasting blood glucose was 16.0 mmol/L, the patient was diagnosed with “type 1 diabetes mellitus” and treated with insulin. At the age of 11 years, slow growth appeared, and the patient’s height was shorter than that of the patient’s peers. Visual loss, urinary frequency, and urinary incontinence were observed within two years. The patient’s parents were consanguineous in marriage (II4, II5) and the grandfather (I1), grandmother (I2), uncle (II1), and uncle (II3) have diabetes. The patient had a BMI of 18.3 kg/m2, HbA1c 14.9%, hydronephrosis, and bilateral ureal expansion. The indirect water deprivation test result was negative, and pituitary MRI suggested a pituitary microadenoma. In addition, there were refractive errors in both eyes and sensorineural deafness in the right ear. Diagnosis of WS was suspected. The genetic testing identified the presence of a homozygous missense mutation of the WFS1 gene, the c.C529T p.R177C. Both parents, a second uncle, and a grandmother were heterozygous carries. Interestingly, the younger brother and sister carried the homozygous mutation, but no relevant clinical findings were found and the time of this study and they were still in close follow-up.

#### Case 3

A 17-year-old male experienced thirst and polyuria over the past 14 years. At the age of 3 years, the patient developed polyuria with a random blood glucose of 42.8 mmol/L. The patient was diagnosed with “type 1 diabetes, diabetic ketosis.” At nine age of 9 years old, the patient’s height was shorter than that of the patient’s peers, and vision and hearing loss decreased at the age of 14 years. The patient’s parents were consanguineous in marriage (III2, III3) and the grandfather (II3), grandmother (II2), and aunt (III6) have diabetes. The patient was 1.3 m tall with a weight of 27 kg, HbA1c of 10.6%, optic nerve atrophy, mild hydronephrosis in the left kidney, and expansion of the upper ureteric segment. WS was suspected and genetic testing identified two homozygous mutations of the WFS1 gene, the c.C529A p.R177S and the c.G2105A p.G702D. The patient’s parents, brother, sister, grandmother, grandfather, sister, and uncle carried homozygous or heterozygous mutations.

#### Case 4

A 17-year-old male noticed polydipsia and polyuria for the past 12 years. At the age of 15, the patient had a random blood glucose of 33.1 mmol/L, and was diagnosed with “type 1 diabetes, diabetic ketosis,” and was discharged after insulin treatment. In recent years, the patient experienced repeated palpitations, sweating, hunger, handshaking, and relief from eating. The parents denied consanguineous marriage or family history of diabetes. The patient was 1.55m tall with a weight of 46 kg, HbA1c level of 10.6%, and mild water accumulation in both kidneys and ureter. WS was suspected and genetic testing identified a homozygous mutation of *WFS1* c.C1885T p.R629W; both parents carried heterozygous mutations.

#### Case 5

A 30-year-old male noticed a gradual decrease in vision in both eyes over the age of 16 years. At the age of 14, the patient was diagnosed with “optic atrophy,” and 2 years later, was diagnosed with “type 1 diabetes” due to fasting blood glucose of 18.0 mmol/L. The patient’s parents denied consanguineous marriage. The patient’s uncle (II2) has diabetes, and two sisters (III4, III6) were also clearly diagnosed with WS. The patient had fasting blood glucose of 17.8 mmol/L, 2 h postprandial blood glucose 20.28 mmol/L, HbA1c 7.9%, fasting C peptide 2.25 ng/ml, 2 h C peptide 2.76 ng/ml, and negative islet-related antibodies. A diagnosis of WS was suspected and genetic testing identified the presence of compound heterozygous mutations, the c.1859_1860del p.V620Gfs * 91 and the c.G2020A p.G674R, of the *WFS1* gene; the patient’s parents and uncles carried heterozygous mutations, and both sisters carried compound heterozygous mutations.

#### Case 6

A 12-year-old male noticed thirst, drinking, polyuria, and wasting over the past 7 years. At the age of 5 years, the patient was diagnosed with type 1 diabetes; and blood glucose level was unknown, and was discharged after insulin treatment. The patient’s parents were consanguineous in marriage (IV3, IV4) with diabetes, and the patient’s brother (V6) was also diagnosed with WS. The patient was 1.4 m tall with a weight of 32 kg, HbA1c of 11.1%, optic nerve atrophy, and refractive error (both eyes). The patient’s brother is 8 years old, after a perfect examination, was diagnosed with WS and given insulin injection treatment. WS was suspected and genetic testing identified homozygous WFS1, c.C529A p.R177S, and c.G2105A p.G702D mutations; both parents, sister, and brother carried compound heterozygous mutations.

#### Case 7

A 19-year-old female noticed thirst, polydipsia, and polyuria 14 years prior to presentation. The local hospital diagnosed “type 1 diabetes” and the patient was given insulin injections. The patient had no history of menstruation. The patient’s parents were consanguineous in marriage (III3, III4) and the younger brother (IV2) was also clearly diagnosed with WS. The patient was 1.23 m tall with a weight of 25 kg and no secondary sexual characteristics. Relevant examination revealed low hypogonadism (LH 0.59IU/L, FSH4.34IU/L, E2 < 10.08Pg/ml) and central hypothyroidism (T4 FT411.02PMOL/L, TSH3.8). In addition, the patient’s anti-islet cell antibody test results were negative, and had severe hydronephrosis and ureteral dilatation. No significant hearing or eye diseases were observed. The patient’s younger brother (IV2) was diagnosed with T1DM at the age of 10 because of abnormal blood glucose levels and was treated with insulin. The patient had cleft lip and palate, and finger and toe development deformities. The patient also had severe hydronephrosis and poor vision. Diagnosis of WS was suspected and genetic testing identified the homozygous mutation of *WFS1*, c.G1393C p.A465P; the patient’s father carried the heterozygous mutation and did not retain the samples.

### Genetic analysis

This study reported seven families with WS and 11 patients screened for a total of 10 mutations in the *WFS1* gene screened. Among these, only c.G1393C showed a VUS mutation, and the rest were pathogenic/likely pathogenic (c.1314_1317delCTTT, c.C529T, c.C529A, c.G2105A, c.C1885T, c.1859_1860del, c.G2020A, c.C529A, and c.G2105A) (Table 1). Among them, except for c.C529T in family B, c.G2105A in family C, c.G2020A in family E, and c.G2105A in family F were reported (without functional verification), the remaining mutation sites have not been reported and belong to new mutations.

**Table 1:** *WFS1* mutations in seven families with Wolfram syndrome

Case	Sex	Age	Clinical manifestation	Accessory examination	fam. hist.	Exon	Nucleotide change	Amino acid change	Hom/Het	Type of mutation	ACMG	First description
1	F	21	Diabetes at the age of 7, and Hyperuria occurred at the age of 11	double hydronephrosis, bilateral ureteral dilatation, optic nerve papillary atrophy in both eyes.	None	8	c.1314_1317delCTTT	p.F438Lfs*3	Hom	Frameshift	LP(PVS1_Strong + PM2 + PP4)	Novel
2	M	14	Diabetes at the age of 10, slow growth at the age of 11, diminution of vision and incontinentia urinae.at 12	hydronephrosis, bilateral ureal expansion, sensorineural deafness	Parents were consanguineous in marriage. Grandpa, grandma, uncle, and uncle have diabetes	5	c.C529T	p.R177C	Hom	Missense	LP(PM3_Supporting + PM1 + PM2 + PM5 + PP3)	[12]
3	M	17	Diabetes at the age of 3, slow growth at the age of 9, diminution of vision at 14	optic nerve atrophy, mild hydronephrosis in the left kidney, and expansion of the upper ureteric segment	Parents were consanguineous in marriage, and grandpa, grandma and aunt have diabetes.	8	c.C529A	p.R177S	Het	Missense	P(PM1 + PM2 + PM5 + PP1_Strong + PP3 + PP4)	Novel
						8	c.G2105A	p.G702D	Het	Missense	P(PM3_Moderate + PM1 + PM2 + PM5 + PP1_Strong + PP3 + PP4)	[13, 14]
4	M	17	Diabetes at the age of 5	optic atrophy, mild hydronephrosis in the left kidney, and expansion of the upper ureteric segment	None	8	c.C1885T	p.R629W	Hom	Missense	LP(PS4 + PM2 + PM3)	Novel
5	M	30	Diminution of vision at 14, diabetes at the age of 16	optic atrophy	Parents denied consanguineous marriage. Uncle have diabetes, and two sisters are diagnosed as WS.	8	c.1859_1860del	p.V620Gfs*91	Het	Frameshift	P(PVS1_Strong + PM2 + PM3 + PP1_Strong + PP4)	Novel
						8	c.G2020A	p.G674R	Het	Missense	P(PM3_Strong + PM1 + PM2 + PM5 + PP1_Strong + PP3 + PP4)	[15, 16]
6	M	12	Diabetes at the age of 5	Optic nerve atrophy, and refractive error (both eyes).	Patients were consanguineous in marriage with diabetes, and his brother was also clearly diagnosed as WS.	8	c.C529A	p.R177S	Het	Missense	LP(PM1 + PM2 + PM5 + PP1_Supporting + PP3 + PP4)	Novel
						8	c.G2105A	p.G702D	Het	Missense	LP(PM3_Moderate + PM1 + PM2 + PM5 + PP1_Supporting + PP3 + PP4)	[13, 14]
7	M	19	Diabetes at the age of 5	No menstruation	Parents were consanguineous in marriage, and the younger brother was also clearly diagnosed as WS	8	c.G1393C	p.A465P	Hom	Missense	VUS(PM2 + PM3_Supporting + PP4)	Novel

P = Pathogenic, LP = Likely Pathogenic, VUS = Uncertain significance

## Discussion

Wolfram syndrome (WS) is a rare, autosomal recessive genetic disorder. It is primarily characterized by childhood- and adolescent-onset diabetes, and progressive optic atrophy. It usually involves multisystem diseases, including diabetes insipidus (DI), diabetes mellitus (DM), visual atrophy (OA), and deafness (D), and is therefore also known as DIDMOAD. Some patients present with growth retardation, hydronephrosis, hypothyroidism, sleep disturbances, and neurological symptoms [4]. The clinical course of WS is characterized by different symptoms in chronological order, with non-immune insulin-dependent diabetes mellitus generally starting at the age of 6; optic atrophy occurring at around 11 years, diabetes insipidus usually occurring at around 14 years, and sensorineural deafness gradually developing during late puberty [5].

The current study showed that the *WFS1* gene is a key pathogenic gene in WS, and the dysfunction or deletion of its encoded protein caused by the mutation of this gene is a major factor in disease occurrence. Approximately 90% of patients are Wolfram syndrome type 1 (WS1) caused by mutations in the *WFS1* gene [6]. The WFS1 protein includes 890 amino acids, and according to The Human Gene Mutation Database, 464 mutations of different types of WFS1 have been reported [7]. This complicates the establishment of clear genotype-to-phenotype correlations. The mutation sites found by the research group are distributed in the C-terminus (endoplasmic reticulum [ER] lumen), N-terminus (cytoplasm), and transmembrane region (ER membrane) of the protein.

The clinical-genetic data of 412 cases of WS in 49 studies showed that 178 mutations of *WFS1* gene (N-terminus, C-terminus, and transmembrane region) were identified in 337 cases, and among them, 35% of missense mutations, 21% of frameshift mutations, 25% of nonsense mutations, 13% of box insertion/deletion mutations, and 2% of shear site mutations, mainly located in the transmembrane domain of Wolframin (encoded by the *WFS1* gene) protein, 94–237 amino acids at the N-terminus, and 100 amino acids at the C-terminus, suggesting that these regions play a key role in the normal functioning of the protein. The clinical phenotypic penetrance time of patients with different genotypes was significant, among which diabetes mellitus and diabetes insipidus phenotypes were the most significant. The WFS1 protein is highly expressed in islet endothelial cells [8], and genome-wide association studies have found that *WFS1* is an important susceptibility gene for diabetes. Decreased cell function of β islets is key to the development of diabetes. The latest study found that the proinsulin in the islets of WFS1-deficient mice accumulates abnormally in the ER; WFS1 directly binds to vesicle cargo proteins (including proinsulin) through the C-terminus of the ER lumen, acts as a transport receptor for vesicle cargo proteins, and its pathogenic mutation located at the C-terminus of the ER lumen hinders the transport of proinsulin to the Golgi apparatus for processing, disrupting insulin secretion and leading to diabetes [9]. Previous studies have focused on the regulation of ER homeostasis by *WFS1*. In primary islets with *WFS1* gene deficiency, the three subpathways upstream of ER stress (PERK, IRE1, and ATF6) are activated and apoptosis, a marker of caspase-3, increases, while cell proliferation is impaired, ultimately leading to impaired islet β cell function and apoptosis [10]. WFS1 mutation or deletion can activate ER stress in pancreatic islet β cells, resulting in impaired β cell function, suggesting that WFS1 helps protect β cells from ER dysfunction [11]; however, the molecular mechanism by which *WFS1* point mutations induce the ER stress response is unclear.

According to previous results, the ER localization of the WFS1 protein suggests that it may have physiological functions, such as membrane transport, secretion, and processing. At the same time, *WFS1* can also be colocalized with secretory granules in β cells, suggesting that it may be involved in the regulation of insulin secretion in β cells. Our team has studied one of the new homozygous double mutation sites (R177S) and (G702D) of *WFS1* and found that the mutations located at the C and N terminals both cause increased *WFS1* transcription and WFS1 protein degradation, proving that mutation sites affect the normal expression of *WFS1*. Future studies will further analyze how point mutations in different regions of WFS1 alter the physicochemical properties of WFS1 protein, cause loss of protein function, clarify the pathogenic mechanism of *WFS1* clinical mutation sites, and help in the pathogenesis of diabetes and the study of new drug targets.

WS is a monogenic disease whose characteristics make it a useful model for understanding complex diseases, such as DM, OA, HD, and other neurologically relevant clinical symptoms [8]. Family cases provide the best resources to study the effects of mutations in different regions on protein function and phenotypic-genotype relationships. *WFS1* mutation causes loss of protein function, but the specific mechanism is unknown. It is worth investigating whether it affects function by affecting protein structural stability and conformational or modification changes.

**Fig. 2:**
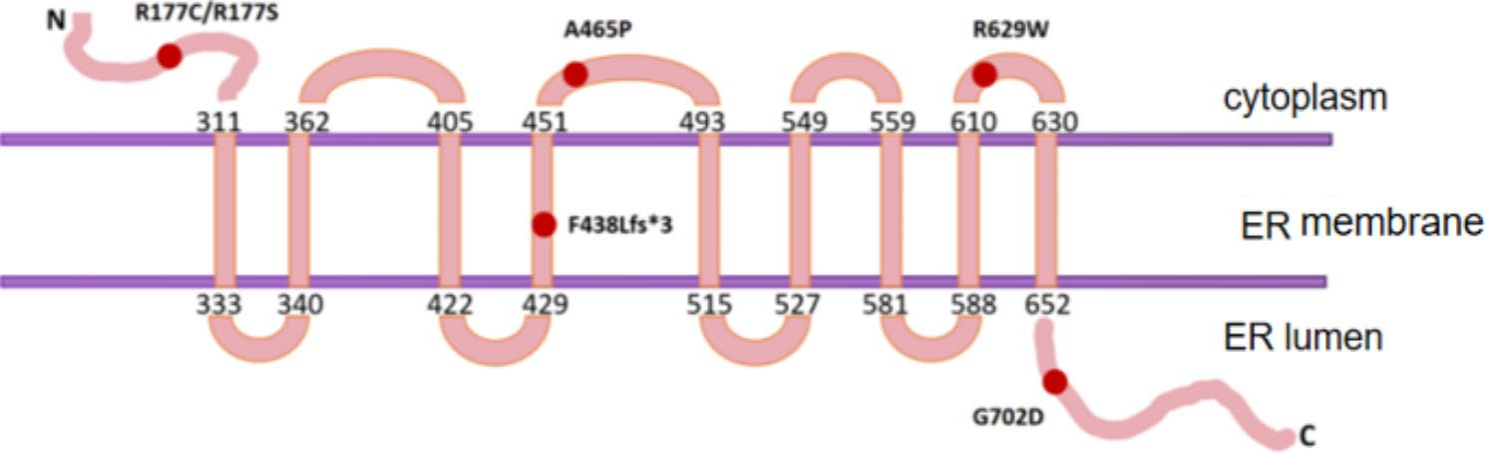
WFS1 protein mutants from seven Wolfram syndrome families

In this study, eight homozygous mutations (five new mutations) were identified in seven families, including one double homozygous mutation (R177S + G702D). Rare double homozygous missense mutations in *WFS1* base were found in family C and F probands, which altered the protein Arg177Ser for GC528_529delinsTA, occurring at the protein N-terminus of the cytoplasm, and another G2105A mutation causes changes in the protein p.G702D, which occurs at the C terminus of the protein in the ER lumen. Other family members developed compound heterozygous mutations at these two sites; however, no relevant symptoms were observed. Therefore, double homozygous mutations may be involved in WS. Four-base deletions of c.1314_1317delCTTT in the family A proband caused p.F438Lfs * 3, which is the cause of patient diabetes, optic nipple atrophy, and diabetes insipidus, but hearing and development are not abnormal; we suspect that this part of the truncated protein may exercise part of the protein function, and the specific reason for this is not clear. A homozygous missense mutation in the proband of family D and the patient’s younger brother, c.C1885T, is now five years old and has diabetes. A c.G1393C homozygous missense mutation in the family G proband caused changes in the protein p.A465P, a locus occurring in the transmembrane region in the cytoplasm, where the proband, the same genotype as the patient’s brother, developed a severe WS phenotype. In conclusion, we found that in seven families of patients with different gene phenotypes (Fig. 2), one mutation caused protein truncation; five missense mutations caused amino acid changes; and different protein regions, N- and C-terminal and transmembrane regions, and cellular localization were the best cases for the phenotype-genotype.

## Conclusions

Wolfram syndrome is a rare autosomal recessive disease that accumulates multiple systems throughout the body, often with diabetes as the first symptom in childhood and adolescence, with a high rate of death and disability and misdiagnosis, and early accurate diagnosis and comprehensive management are the key. The main causal gene of Wolfram syndrome is *WFS1*, at present, we have collected clinical phenotypes, but it is not clear whether these mutants are directly involved in the development of the disease and the relationship between these mutations and the phenotypes. In future studies, focusing on the effects of different types of mutants on the function of the WFS1 protein and will reveal the link between different mutation sites in the pathogenesis of Wolfram syndrome. In addition, the family line of Wolfram syndrome will be followed up to evaluate the relationship between point mutations in different regions of WFS1 and the severity and spectrum of the clinical phenotype, which will lay a foundation for the precise clinical diagnosis and treatment of Wolfram syndrome.

## Methods

### Ethics statement

The study was reviewed and approved by the Hospital Ethics Committee (KY2022102801) and all study participants or their guardians signed an informed consent form. Phenotypic evaluation was completed through a review of medical records and in-person examinations by physicians who were co-investigators in the study.

### Exome sequencing and variant analysis

Mutation screening: Exons were captured by Exome Research Panel v2.0 + Exome Research Panel v2.0 and the Illumina sequencing platform for high-throughput sequencing.

Gene data analysis: The sequencing data were aligned to the human genome using BWA (0.7.12-r1039) software, and the mutation sites were annotated using dbSNP, Clinvar, ExAC, thousand human genome by Hannover ($ d a t e, 2015-06-17). The suspected pathogenic mutations were graded according to the American Society of Medical Genetics and Genomics (ACMG) Genetic Variant Grading System. Simultaneously, software prediction and population database annotation were performed for unreported mutations or mutations with unclear clinical significance.

Verification of the suspected pathogenic mutation: After PCR, the target sequence of the suspected pathogenic mutation was verified by ABI3730 Sanger sequencing, and the sequencing results were verified using DNASTAR subroutine SeqMan software.

## Data Availability

All patient data has been anonymized, and any further information may be obtained from the corresponding author.

## Abbreviations

WS: Wolfram syndrome
*WFS1*: Wolfram syndrome1
*CISD2*: CDGSH iron-sulfur domain-containing protein 2
ER: Endoplasmic Reticulum
